# Immunotherapy of Brain Cancers: The Past, the Present, and Future Directions

**DOI:** 10.1155/2010/296453

**Published:** 2011-03-08

**Authors:** Lisheng Ge, Neil Hoa, Daniela A. Bota, Josephine Natividad, Andrew Howat, Martin R. Jadus

**Affiliations:** ^1^Pathology and Laboratory Medicine Service, Department of Diagnostic and Molecular Medicine Health Care Group, VA Long Beach Healthcare System, 5901 E. 7th Street, Long Beach, CA 90822, USA; ^2^Department of Neurology and Department of Neurological Surgery, Chao Family Comprehensive Cancer Center, UC Irvine School of Medicine, University of California, Irvine, CA 92697, USA; ^3^Chao Family Comprehensive Cancer Center, UC, Irvine School of Medicine, University of California, Irvine, CA 92697, USA; ^4^Pathology and Laboratory Medicine, University of California, Irvine, CA 92697, USA

## Abstract

Treatment of brain cancers, especially high grade gliomas (WHO stage III and IV) is slowly making progress, but not as fast as medical researchers and the patients would like. Immunotherapy offers the opportunity to allow the patient's own immune system a chance to help eliminate the cancer. Immunotherapy's strength is that it efficiently treats relatively small tumors in experimental animal models. For some patients, immunotherapy has worked for them while not showing long-term toxicity. In this paper, we will trace the history of immunotherapy for brain cancers. We will also highlight some of the possible directions that this field may be taking in the immediate future for improving this therapeutic option.

## 1. Introduction

Immunotherapy for cancer has made progress and is now becoming part of the treatment options that are more frequently discussed with oncology patients. Previously, this type of treatment was given to patients with advanced disease, with only a few months to live. Needless to say, the final results were often disappointing. While the failures told us what strategies did not work, it showed that immunotherapy was generally safe and did not immediately kill the patient. It also showed that the dreaded autoimmunity was not being induced. These results also spurred the development of different approaches, after better understandings of cancer immunology were unexpectedly discovered. This illustrates our need to learn more about basic *in vivo* cancer immunology before clinical therapies can be fully predicted. The proper timing and use of the right antibodies or cells has also allowed this progress to occur. The herceptin antibody targeting the her2/neu proto-oncogene has benefited those women with breast and ovarian cancers that overexpressed this receptor. This discovery showed that targeting a cell-surface receptor controlling a key biological function, as opposed to any available tumor surface antigen, was the key to generating useful clinical responses. Recently, PROVENGE marketed by Denderon Corp, was given FDA approval in the USA to treat refractory prostate cancer in men. This prostate tumor-antigen (prostatic acid phosphatase)-granulocyte macrophage-colony stimulating factor fusion protein does stimulate dendritic cells *in vitro*. When these *ex vivo* activated dendritic cells are reintroduced back into the patient, the host's antitumor T cells are restimulated, which subsequently attacks the cancer. This immune response does translate into an additional four months of life. These two success stories demonstrate that progress towards cancer is slowly advancing and we eagerly await more successes as the overall field continues to advance and mature.

Glioblastoma multiforme (GBM, WHO stage IV) and anaplastic astrocytomas (WHO stage III) are aggressive and lethal cancers. These cancers are almost always fatal within five years (2010 Central Brain Tumor Registry). These tumors are very invasive; this contributes to their resistance to be cured by traditional surgical resection and directed radiation therapy. Hence the need to develop better therapies still exists. The advantage of generating an immune response towards a cancer is that the immune effectors (cells or antibodies) can now seek out and destroy the tumor cells that are located in inaccessible sites that traditional surgery, radiation, or chemotherapeutic drugs cannot reach.

Due to the relative isolation from the systemic circulation, because of the blood brain barrier, the initiation of productive immune responses in the brain is more limited than other types of cancers [1]. Local microglial cells can process and present tumor-associated antigens to T lymphocytes [2–5]. However few naïve T cells normally transit into the brain. Normal brain cells also express Fas Ligand and express TGF-*β* [6, 7], making immune responses harder to be sustained. Hence lymphoid cells must be recruited from the periphery by a variety of cytokines and chemokines. Once effector lymphocytes infiltrate the tumor, they can mediate antibrain tumor immunity. Despite these obstacles, progress is slowly being made in neuro-onco-immunotherapy. Unless some extraordinary discovery is made, immune-based therapies must be combined with other modalities that target other critical aspects of cancer biology. This paper will focus on the natural progressions that are leading us towards successful immunotherapy for brain cancers.

## 2. Types of Immunotherapy

Immunological-based treatments have been used in several ways to treat cancer. These include (1) nonspecific methods using adjuvants, lymphokine activated killer cells, or gene-modified tumor cells; (2) specific immunotherapy include using monoclonal antibodies, tumor infiltrating lymphocytes, allogeneic reactive T cells, chimeric antigen-redirected T cells, purified and cloned tumor antigens used either alone or in combination with *in vitro* cultured dendritic cells (DCs).

### 2.1. Nonspecific Approaches

#### 2.1.1. Adjuvants

Nonspecific approaches include using natural adjuvants such as bacillus Calmette-Guérin (BCG, *Mycobacteria bovis*), muramyl dipeptide (MDP), Detox (lipopolysaccharide with lipid A removed). Janeway [8] once wrote: “adjuvants are the immunologist's dirty little secret,” in that these molecules are needed to provoke the immune response. Adjuvants work by stimulating local antigen presenting cells such as dendritic cells, macrophages, and B cells via toll-like receptors and pathogen-associated recognition molecules. TLR receptor stimulants as imiquimod, polyinosinic-polycytidylic acid stabilized with polylysine and carboxymethyl-cellulose (Poly ICLC), CpG containing DNA, and other synthetic molecules are used to stimulate dendritic cells, which activate either cell-mediated or humoral immunity provided the antigens of choice are also present. 

In some parts of the world, BCG is used as a prophylactic vaccine to stimulate immunity towards *Mycobacteria tuberculosis,* due to its very strong immunogenic properties, as well as common antigenic determinants. A purified protein derived (PPD) from *M. tuberculosis *is the difference between Freund's complete and incomplete adjuvant commonly used in antibody production in animals. BCG is also used in a therapeutic vaccination setting to actively treat human bladder cancer [9]. Here the initial nonspecific inflammation in response to BCG injected directly into the tumor leads to an innate immune response that causes tumor cell death. After the tumor dies it is followed by lasting cellular immunity towards the bladder cancer. Wikstrand and Bigner [10] used BCG and human glioma cells to generate good antibody responses towards the glioma cells without any signs of autoimmunity. Albright et al. [11] used BCG to treat GBM patients in 1976. Here 10^7^ BCG organisms were used as an intradermal stimulus to induce delayed type hypersensitivity (DTH) reactions. Their patients were subsequently injected with autologous glioma cells with the purified protein derivative (PPD). The theory here was that when the host mounted the recall response towards the PPD, the glioma antigens would be incorporated in this DTH response and mount a primary immune response towards the glioma cells. Once these antibodies and immunized lymphocytes were elicited, they would then home into the brain tumors and mediate their antitumor effects. Unfortunately, this therapy failed to achieve much inflammation within the relapsing glioma along with no improved patient survival. The use of adjuvants was largely abandoned, until its ability to stimulate dendritic cell maturation via different receptors was recently discovered (see dendritic cells).

#### 2.1.2. Natural Adjuvants: Heat Shock Proteins (HSP)

Heat shock proteins are induced by a variety of stressful conditions: heat, radiation, chemotherapy, nutrient starvation, hypoxia, and so forth. These molecules are responsible for assisting in the synthesis and correct folding of newly synthesized proteins, thereby replacing the stress-damaged proteins. 

The HSPs were described as “natural adjuvants,” since they provoke immunity [12]. Several of these HSPs: HSP70 and gp96 (also known as GRP94) were identified as tumor-specific antigens [13]. In other cases, HSP70, HSP90, and gp96 increased the immunogenicity of tumors by improving T-cells immune responses [14, 15]. Proteins synthesized by tumor cells include potential antigens. As these antigens are degraded by the proteasome, it was reported that HSPs acted as chaperones [14, 15]; these molecular chaperones then shuttle these antigenic peptides to the endoplasmic reticulum, where they can be eventually loaded onto the MHC. Tumor cells can present HSPs on their surfaces (our unpublished data), release these peptide/HSP complexes, presumably via exosomes [16–18]. Here the host's antigen presenting cells (APCs), both dendritic cells and macrophages, can take up these antigens/HSPs via the CD91 receptor [19]. Peptides complexed to HSPs stimulate better immune responses than when the antigenic peptide is not complexed to the HSP [13]. HSP70 and gp96 also have been reported to enhance dendritic cell maturation [20, 21]. HSP70 also acts as a cytokine, which stimulates tumor necrosis factor, IL-1 and IL-6 production from CD14+ monocytes [22]. These CD14+ cells also include the immature dendritic cells. Thus, HSPs might be able to stimulate immune responses via its natural adjuvant activity, while simultaneously delivering the antigenic peptides provoking specific immune responses. 

On the clinical level, Antigenics, Inc. (Lexington, MA) has developed the technology to produce a clinical product called Oncophage. Here the surgically resected tumor is taken and the gp96 component (HSPPC-96) from the tumor is isolated. This purified gp96 (presumably binding antigenic peptides) is then formulated as a custom-made vaccine using the GS-21 adjuvant for each patient. This approach has been given fast track and orphan drug designations by the US FDA and the EMEA for a couple of cancers. At the University of California, San Francisco, Oncophage is being used in Phase 2 trials to treat GBM. At the recent International Conference on Brain Tumor in Travemunde, Germany (May 2010), it was reported that of the 32 evaluable patients with recurrent GBM given Oncophage, 41% survived up to a year or longer [23]. The authors also saw a robust immune response within increased Th1 cytokine production by the immunized T cells.

#### 2.1.3. Lymphokine Activated Killer Cells (LAK)

LAK cells are NK and NK-T like (CD8+) cells that when stimulated with lymphokines (cytokines) like interleukin-2 (IL-2) become nonspecific tumoricidal cells. LAK cells kill most, if not all, tumor cells quite well *in vitro* in a non-MHC restricted manner. When IL-2 or interferon-*γ* (IFN-*γ*) transduced fibroblasts were coinjected with the murine GL261 glioma, the glioma cells were rejected by the recruited NK and LAK cells. The LAK cells were activated *in situ* by the cytokines [24]. However in a rat glioma model using the F98 glioma cell line, the recruited rat LAK cells were not as successful as the previous mouse model [25]. The clinical application of LAK cells has been effective only towards some melanoma and renal cancers [26]. Occasionally a response towards a human glioma is seen [27, 28]. Hoag Hospital in Newport Beach (California) is currently using LAK cells that are implanted into their patient's brain tumor cavity after surgery [29, 30]. The main disadvantage of LAK cells, is that they release multiple cytokines (IFN-*γ*, tumor necrosis factor-*α* (TNF-*α*)), which cause many of the unwanted pharmacological toxicities associated with this clinical therapy.

#### 2.1.4. *γδ* T Cells

Normally the T cells that we think about, are those T cells with the classic *αβ* T-cell receptor (TCR) rearrangements. These cells normally circulate through the blood and reside in the lymph nodes and spleen. These cells reside in many tumors as the tumor-infiltrating lymphocytes (TILs). But another cell type also goes through the same thymus-education pathway, except that these cells utilize their rearranged *γδ* T-cell receptors to recognize their antigens. These *γδ* T-cell receptors are more restricted in their TCR diversity and are not MHC restricted, although they may recognize nonclassic HLA-E and HLA-G molecules. These lymphocytes were initially discovered to be cytotoxic towards leukemia cells, but Fujimiya and colleagues [31] discovered that these cells also had the ability to recognize and kill glioma cells *in vitro*. Several of the ligands that *γδ* T cells can recognize tumor cells (MICA, MICB, and UL-16 binding proteins) are also found on gliomas [32]. In the United States, Lamb and colleagues [33, 34] confirmed the previous study. Their human *γδ* T cells failed to kill normal astrocytes. They also discovered that the *γδ* T cells can be expanded in the presence of low doses of IL-2 and zoledronic acid, so that sufficient number of cells could be generated for infusion back into patients. Human *γδ* T cells when implanted into nude mice showed immunological efficacy against U251 xenografts [35]. This non-MHC restricted killing by *γδ* T cells opens up the possibility that allogeneic donors could be used for therapeutic purposes in gliomas without risking the possibility of graft-versus-host reactions or autoimmune diseases. Clinical trials using this approach against brain cancers are expected to begin in the summer/fall of 2011 at the University of Alabama, Birmingham.

#### 2.1.5. Gene Therapy

Gene therapy using various cytokines and costimulatory molecules was used in experimental glioma models to induce stronger immune responses. IL-2 and IFN-*γ* transduced rat RG2 (also known as D74) glioma cells, when injected into the brains of naïve rats, resulted in premature death of the rats due to changes in the vasculature of the brain [36]. Peripheral vaccination using N32 rat glioma cells transduced with IFN-*γ* and interleukin 7 induced intracranial rejection of the parental N32 glioma [37]. The membrane form of macrophage colony stimulating factor (mM-CSF) as opposed to the soluble form of M-CSF, when transduced into T9 (also known as 9L) glioma cells caused the transduced cells to be immediately rejected [38–40]. After glioma rejection occurred, tumor immunity was concurrently induced. These rejected mM-CSF positive cells, not only lead to excellent prophylactic vaccination, but could also be successfully combined with antiangiogenic therapy to therapeutically treat seven-day established intracranial gliomas [41]. Granulocyte-macrophage colony stimulating factor (GM-CSF) and interleukin-4 (IL-4) transduced 9L gliomas also led to tumor immunization under similar conditions [42, 43]. Since GBM patients relapse so fast, it was considered unlikely, that one could establish the patient's primary glioma cell line and then transduce them with immunostimulatory molecules or cytokines fast enough, before the glioma relapse occurs. Thus this genetic approach using autologous gliomas has not been used for neuro-oncology.

One limitation of using GM-CSF transduced glioma cells as a tumor vaccine is that some human gliomas make and use GM-CSF as a potential autocrine growth factor [44, 45]. So this cytokine must be carefully used, so as not to enhance the growth of the primary glioma. One way to avoid any possible GM-CSF-dependent autocrine pathways by gliomas themselves is to make use of the versatility of the APC such as dendritic cells (DCs) in an *ex vivo* setting using the recombinant cytokine (see below). This way this recombinant cytokine does not directly interact with the glioma, while still mediating its therapeutic beneficial effects.

### 2.2. Antigenic Specific Pathways

#### 2.2.1. Antibody Approaches towards Glioma's Vasculature

Angiogenesis is crucial for tumor growth greater than 1-2 mm^3^. Multiple growth factors and proteolytic enzymes play different roles in angiogenesis, but which pathway is the most critical one for any given tumor is still actively debated. Mostly likely, several antiangiogenic agents are needed to target multiple sites, simultaneously to shut down this entire process. Since gliomas are highly vascularized, these antiangiogenic approaches have a potential to work, assuming the right glioma and its angiogenic pathway can be selected. Based upon microarray data analysis [46, 47], gliomas are classified into at least three subtypes: classical, proneural, or mesenchymal. Each form has its own unique characteristics and survival rates. This data may allow for better targeting of these types of glioma, once these angiogenic pathways are identified for that individual glioma. We will discuss only the antibodies that are currently being tried against brain cancer angiogenesis. A number of small, cell-permeable, receptor tyrosine kinase inhibitors are being used clinically against brain tumor angiogenesis, but we will not discuss them here, since they are not immunologically based. For further references see the current reviews by [48–50]. There is evidence that some antiangiogenic drugs can be successfully combined with a tumor vaccine to treat an experimental one-week established intracranial glioma [41].

**Antivascular Endothelial Growth Factor (VEGF) Pathways**

VEGF was the first cytokine to be associated with tumor angiogenesis. There are four forms of VEGF: VEGF-A, VEGF-B, VEGF-C, and VEGF-D. These growth factors bind to two specific VEGF receptors, types 1 and 2. The Avastin (bevacizumab) antibody binds and neutralizes the VEGF and prevents the cytokine from properly stimulating the VEGF receptors. Theoretical interfering with this pathway should prevent the endothelial cell precursors from being recruited into the growing tumor, thereby blocking early angiogenesis. There is some association with VEGF-driven pathways with the mesenchymal type of GBM. Avastin along with different chemotherapeutics did give high response rates in recurrent gliomas ranging from 43–63% [51–53]. This combined approach using an antiangiogenic antibody with chemotherapeutics could improve the efficacy of treatment, even if Avastin alone has some antiglioma effect [54]. As a consequence, Avastin has been registered by the FDA in May 2009 for treatment of relapsing GBM after standard treatment.

Another antibody is ramucirumab; this antibody targets the VEGF Receptor 2 [55]. This antibody (IMC-1121B) is being developed by Imclone Systems. In Phase I studies using a variety of solid tumors (no glioma patients were tested), systemic serum VEGF-A levels did rise during the therapy. Tumor perfusion and vascularity were diminished in most of the patients that received the ramucirumab as predicted [55]. For GBM therapy, it would seem that both antibodies towards VEGF and VEGFR2 could be used together to completely inhibit this VEGF-mediated pathway.

**Antihepatocyte Growth Factor/Scatter Factor (HGF/SF)**

As its name implies, this cytokine/growth factor was initially discovered in liver cancers. But gliomas make this protein and use it as an autocrine cytokine by binding to its receptor called c-Met [56]. Upon binding its receptor, HGF/SF is thought to stimulate the invasive behavior of the gliomas. Amgen has developed the AMG102 antibody, which has *in vivo* efficacy against human U87 gliomas growing in immunodeficient mice [57]. We are not aware of any current clinical trials being performed for neuro-oncology with this antibody.

#### 2.2.2. Antibodies Directed towards the Glioma

**Antiepidermal Growth Factor Receptor (EGFR) Antibodies**

EGFR is a predominant pathway that helps characterize the classical/proliferative type of gliomas. These receptors bind either to EGF or transforming growth factor-*α* (TGF-*α*). These receptors can either be mutated or overexpressed due to genetic amplifications. The most common mutation of the EGFR is the EGFRvIII mutation, caused by a deletion of 268 amino acids in the extracellular region that constitutively activates this receptor. At least 2 different antibodies towards this receptor are currently used for clinical studies: Cetuximab/Erbitux [58] and Nimotuzumab [59]. A preclinical model showed promise in immunodeficient mice [59], but this success was not observed in clinical trials [60].

**Antiplatelet Derived Growth Factor Receptor *α* (PDGFR*α*) Antibody**

The PDGFR*α*-mediated pathway is representative of the proneural subclass of GBM. This receptor can bind either to PDGF-AA, PDGF-AB, PDGF-BB, or PDGF-CC [61]. Again, PDGFR is amplified and overexpressed in some GBM. The IMC3G3 antibody is being explored as a clinical therapy for this receptor. Cytomegalovirus (CMV) has been linked with human GBM [62] (see viral antigens). CMV is reported to use the PDGFR*α* as an attachment factor [63]. This antibody may be clinically significant, because it inhibits growth factor stimulation of the glioma along with interfering with CMV infections. One other advantage of targeting the PDGF receptor is that it can also target the pericytes/fibroblasts which give structural support to the endothelial cells [64]. This antibody may also interfere with the antiangiogenic pathway, too.

**Tenascin C**

Tenascin C is a glycoprotein specifically made by gliomas. It is laid down as an extracellular matrix. Monoclonal antibodies towards tenascin-C (clone 81C6) [65] by Duke University or the BC-2 and BC-4 clones used at Bufalini Hospital (Cesena, Italy) [66, 67] have been developed and are capable of localizing to various GBM in patients. Attempts have been made to use radioisotope (I^131^, Y^90^ or At^211^)-conjugated antibodies to treat gliomas. When these antibodies were injected into their respective patients, these antibodies localized to the glioma. In theory, the radiation released from the isotope-labeled antibody should damage and kill the adjacent cancer. To date some successes (stabilized disease) are seen in their respective American and Italian cohorts using these radiolabeled antibodies [65, 66]. These antibodies will probably be quite useful for finding residual pockets of the tumors. Since the tenascin c is not strictly a glioma membrane protein, this may be a limiting factor for the direct glioma treatment, and is probably not the optimal way to treat glioma cells. Another possible problem with this overall approach is that GBM stem cells are resistant to the effects of radiation (see GBM stem cells).

**Bispecific Antibody**

In the 1980's the concept of using bispecific antibodies came into vogue. Here two different monoclonal antibodies are used, the first antibody binds specifically to the cancer while the second antibody binds the T or NK cells (via CD3). The antibodies are selectively reduced, so that a heavy and light chains still remain together as a heterodimer, maintaining the antibody binding specificity. Then the single heavy/light chain from the first antibody is mixed together with an identically prepared second antibody, which binds to the cancer cell's surface. Afterwards, the two heterodimers are allowed to reform their disulfide bonds, producing a stable antibody now with two different antigen-binding specificities. The bispecific hybrid antibody is selected, which simultaneously binds to both cells/antigens. Thus using this bispecific antibody, the effector lymphocyte now physically binds to the cancer cells and helps initiates cytolytic function by the lymphocyte against the cancer.

Nitta et al. [68] used a bispecific antibody towards CD3 and a tumor antigen, originally developed against lung cancer (NE150, 69). But NE150 also had cross-reactive properties towards human gliomas. When they used this bispecific antibody with LAK cells, this group achieved better clinical responses against gliomas (four out of ten patients showed tumor regression within 10–18 months), while all eight patients treated with LAK cells alone showed tumor recurrence. This study using bispecific antibody seemed successful, but it was very labor intensive in the biochemical preparation. It was deemed impractical to generate sufficient antibody to treat multiple patients in order to demonstrate statistical significant improvement in a larger study. But the proof of concept here was established with this study.

**Single-Chain Variable Fragmented Antibodies**

Antibodies have a molecular weight of 150,000 kd, so their ability to penetrate deeply into tumors or tissues is thereby limited. With the advent of genetic engineering, one can take hybridoma cells and isolate the mRNA for the antibody. The variable binding regions of the N terminals (first domains) of the heavy and light chains can be genetically cloned and ligated together to maintain their ability to bind to the antigen. So the term, single-chain variable antibody fragment (scFv), was coined. These recombinant molecules are now only about 25 kd in size, which in theory should be able to penetrate between cells better than normal antibodies. Early studies were used against the EGFRvIII protein [69, 70]. Improved tumor penetrance by this scFv was noted. Recently, this technique has been used with a phage-display technology to produce unlimited amounts of this recombinant protein. Last year, Kuan et al. [71] used this technique to make scFv that target the multidrug resistance protein-3 (MRP3) gene found on gliomas. These recombinant proteins had very good binding affinities for gliomas and could be able to be conjugated with either drugs or radioisotopes. Since this MRP3 specific scFV targets a key biological response (reverse chemotherapeutic drug transporter), this scFV should be combined with chemotherapy to generate synergistic clinical effects.

**Antibodies on the Horizon**

Two antibodies (ipilimumab and daclizumab) are on the horizon, which could have potential impact on glioma immunotherapy. Ipilimumab is the antibody that targets an immunomodulatory molecule, called CTLA-4. When naïve T cells become activated, a late antigen called CTLA-4 is expressed. CTLA-4 then binds and inhibits the CD28 costimulatory pathway. Thus, this molecule naturally represses T cells. By preventing this CTLA-4-mediated downregulation, an enhanced immune response can be sustained and can probably enhance antitumor immune responses. Recently, this antibody has been successfully used for the treatment of melanoma [72]. Here an additional, four months of survival were noted in these patients.

The second antibody is Daclizumab, which targets the high-affinity interleukin-2 receptor-*α* on T cells. This is another potential monoclonal antibody that can improve patient survival by preventing the actions of T-regulatory cells. T-regulatory cells (Treg) are IL-2R*α*+ (CD25) cells and thus more sensitive to the antibody compared to the cytotoxic T cells (see below). By eliminating Treg cells, a more sustained antitumor immune response can also be maintained. Here the idea is to eliminate the Treg before they inhibit the an optimal antiglioma immune response. In experimental models, eliminating Tregs improves therapeutic efficacy of immunotherapy [73]. It will not be long before either of these two antibodies will be combined with some clinical trial to improve glioma therapy.

**IgE?**

An unexpected discovery was initially reported by Wrensch and colleagues [74, 75]. Here atopic patients who frequently suffer from immediate hypersensitivity reactions: hay fever, asthma have a lowered risk of contracting gliomas. Those patients who have high serum levels of IgE and who do develop glioma, statistically survive somewhat longer than those patients with low IgE levels. These studies have been reproduced in a larger meta-analysis and seem highly credible [76]. IgE is the antibody that mediates immediate hyper sensitivities. Nothing is known about how the degranulating basophils and mast cells responding to IgE-mediated cross-linking affect glioma cells or the glioma's vasculature. So this phenomenon could prove to have major repercussions for future glioma therapy. Genetic engineering with some of the antibodies described above could be constructed using the IgE framework. Most IgG antibodies work therapeutically when applied in the milli to microgram range, while immunopharmacological effects of IgE occurs in the nano to picogram range. Thus, these redirected antibodies might have unique properties in achieving clinical effects at lower doses than the IgG-based antibodies. Of course, these proposed studies are very highly speculative and require stringent animal safety tests to assure that animals and then patients do not immediately go in acute anaphylactic shock upon contact with gliomas. Nevertheless, this is a very intriguing concept.

#### 2.2.3. Cellular Approaches

After the nonspecific LAK cell experience in the mid to late 1980's, the next progression of cellular immunotherapy was to use those tumor infiltrating lymphocytes (TIL) and the effector T cells that are found in the local lymph nodes draining the tumor. These T cells were already primed *in vivo* towards the patient's own tumor cells. This methodology was developed in the days prior to our current understanding and routine use of dendritic cells. Here TILs were selectively expanded from either the tumor or draining lymph node cells, by using IL-2, supplemented with LAK-conditioned supernatant as a source of other T-cell immunostimulatory cytokines [77]. By routinely restimulating these cells with irradiated or killed tumor cells, this helped maintain T-cell specificity. When reinfused back into the patients, the CD8+ CTLs had the inherent advantage over the LAK cells, in that these CTLs were capable of killing multiple target cells. CTLs only release their cytokines when properly stimulated by the self-MHC and peptide. These T cells reduced much of the clinical toxicity previously seen with LAK cells. CD8+ CTL were often the cell of choice to examine since their effector function (cytolysis) was easily measured by radioisotope release assays. This TIL/CTL approach again proved to be somewhat better at generating clinical responses to melanoma and renal cancer [77]. 

In rodent models, the use of TILs and draining lymph-node-derived T cells expanded *ex vivo *did prove to be efficacious for the treatment of rodent gliomas [78–80]. Dunn et al. [81] have recently reviewed the history of clinical glioma-based T cells in better details; readers are encouraged to read this article. GBM TILs were successfully used by Quattrocchi et al. [82] to treat their patients, where they took TILs derived from recurrent malignant gliomas and expanded the CD3+ T cell *in vitro* with IL-2. Both CD4+ and CD8+ cells were expanded and then reinfused into the surgical cavity via an Ommaya reservoir. After the infusion of the T cells, the patient was given IL-2 maintenance therapy, three times a week for a month. There was one complete responder (45 months) out of 6 patients treated. Plautz et al. [83] also showed some clinical successes using immunized T cells obtained from inguinal lymph nodes and expanding them with a mitogen [83]. After a short-term *ex vivo* expansion these cells were then reinfused back into the patient.

**Allogeneic Mixed Lymphocyte Reactive T Cells**

Another lymphocyte approach towards brain cancers was pioneered by Kruse et al. [84] and reviewed in Yang et al. [85]. Here lymphocytes derived from histoincompatible allogeneic human blood donors are combined with the patient's irradiated lymphocytes. A mixed lymphocyte reaction sensitizes the allogeneic donor's peripheral blood mononuclear cells towards the patient's MHC. These alloactivated T cells are then expanded in low doses of IL-2 for another 2-3 weeks. These alloactivated lymphocytes are cytotoxic towards the patient's lymphoblasts. When these effector cells are then implanted into the resection cavity, these CTLs can eliminate the remaining glioma cells. This procedure was repeated up to 5 times for each patient. Some long-term (>15 yr) survivors were documented against stage III gliomas [86]. Early concerns that these allospecific CTLs would indiscriminately kill nontumorous host brain cells and induce autoimmunity have been proven to be unfounded. Thus, a larger dose-escalation study using this technique for stage III astrocytomas is open for accrual for 15 patients in the southern California area in collaboration with Dr. Linda Liau (UCLA) to expand and confirm the validity of this therapeutic modality.

**T-Helper Cells**

CD4+ T cells also have important antitumor immune effector functions. CD4+ cells recognize MHC class II restricted peptides. Some CD4+ T cells can kill tumor cells either via Fas Ligand-dependent [87] or perforin-dependent pathways [88]. But most tumors do not express MHC class II antigens, so how antitumor effects are directly mediated by these T cells is not really known. The CD4+ cells' probable mechanism of action involves the release of cytokines and other mediators, which either targets the tumor directly or the tumor's vasculature. The best possibility is that as a result of stimulation by the DC, these CD4+ cells release cytokines (IL-2, IL-6, IFN-*γ*, TNF, lymphotoxin (LT)) that assist in the expansion of the CD8+ CTLs. Some cytokines released from type 2 helper T cells (Th2) can assist in B-cell activation and maturation into making specific IgG antibodies. In the rat 9L (also known as T9) glioma model, effective immunity was seen by the adoptive transfer of immunized CD4+ T cells [39, 89]. Furthermore, Okada et al. [90] showed that rats immunized with IL-4-transduced 9L gliomas did make antibodies against at least three rat glioma-associated proteins, not previously known to be glioma-associated antigens. In a humanized SCID mouse model, CD4+ T cells were isolated from a mouse that was actively rejecting a membrane isoform of macrophage colony stimulating factor (mM-CSF) transduced U251 glioma [91]. But no exact mechanisms were provided in these last studies, explaining how the direct beneficial role of CD4+ T cells occurred in these glioma models other than by the “classic” T-helper cell function.

**T-Regulatory Cells**

Gliomas frequently contain T-regulatory (Treg) cells [92–94] which are CD4+, CD25+ (IL-2R*α*+), and FoxP3+ cells. These cells are probably induced in an effort to maintain immune homeostasis. In the gut, these types of cells also can be induced into becoming follicular helper T cells that assist B cells into making IgA [95]. Because of microenvironmental conditions in the glioma such TGF-*β* and PGE_2_, these T cells are forced to become these suppressor types of cells. These Treg cells inhibit T-cell effector functions; this might account for the failure of GBM-derived TILs to successfully eliminate the glioma in clinical trials. Tregs work in several ways [92–94] to inhibit the necessary antitumor effector mechanism. Methods to eliminate Treg function will likely improve clinical results in future trials. Currently antibodies against the IL-2R*α* (Daclizumab) are used to eliminate these Treg cells. The earnest investigation of Treg in cancer has blossomed in the last five to seven years. So it is unknown what percentage of Tregs was previously expanded as TIL populations and inadvertedly used in previous clinical TIL studies, which most likely failed to treat these patients.

T-reg cells and another type of T-helper cell, called Th17 cells, seem to share a common early stage pathway [96, 97]. Naïve T cells upon exposure to antigen and TGF-*β* can give rise to either Treg or Th17 cells. To get Th17 cells the presence of IL-6 is required. Both cytokines are produced by gliomas. Recently, Th17 cells were described in murine and human gliomas [98], but their beneficial or inhibitory actions was not elucidated. In a mouse model of melanoma, Th17 cells could be used to eliminate very large established tumors [99].

**Redirected T Cells Using Chimeric Antigen Receptors (CARs)**

Generating T-cell clones responding towards tumor-specific antigens either by TILs or by DC restimulated T-cells clones is quite labor-intensive and naturally quite costly. The potential for microbial contaminations and incubator/power failures increases with time. This logistical problem lead to the concept of redirecting T cells or NK cells by genetically manipulating these effector lymphocytes by using man-made chimeric antigen receptors (CARs). The same basic technology described for scFv antibodies can now be married to lymphoid effector cells. Here one splices the scFv region via a spacer region to the transmembrane spanning regions of the CD28 molecule. The intracellular region of TCR*ζ* chains is also ligated into this construct. This artificial receptor, when activated upon proper T-cell surface-binding to the tumor, initiates cytolytic T cell function (release of perforin/granzymes or cytokines). These kinds of genetically engineered receptors have also been called zetakines or T-bodies.

These redirected T cells can recognize a tumor's cell-surface molecule. Other advantages of using CAR constructs are (1) independent of HLA expression where HLA is frequently down regulated or eliminated on the gliomas; (2) can react better to modestly expressed tumor targets; (3) CARs have uniformity and high-degree of expression; and (4) reliably generate T cells in a relatively short time for clinical usage (10–15 days as opposed to 10–12 weeks needed for CTLs).

**Figure 1:**
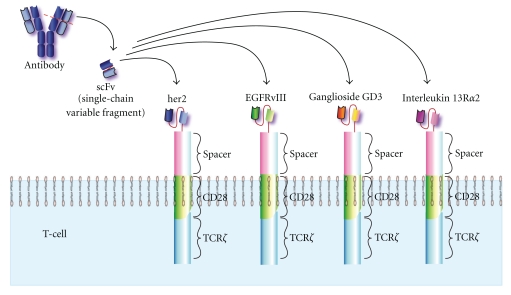
Chimeric Antigen Receptors (CAR) used for potential human therapies of brain cancers. The monoclonal antibody towards either her2/neu, EGFRvIII, ganglioside GD3, or IL13R*α*2 is the initial source of the genetic material. The first domains of the heavy and light chains are ligated together with a short spacer region to create the single chain variable fragment (scFv), to preserve the recombinant proteins' antigen binding region. Another spacer region is ligated from the scFV region to the transmembrane CD28 molecule, followed by the TCR*ζ* chain. After the T cells are transfected with the adenoviral construct, these T cells are then allowed to interact with the tumors. Upon contact with the antigen on the tumor, the CAR is activated and the TCR*ζ* chain is now activated which then stimulates antitumor mediator effector function, that is, cytolysin or cytokine release.

Chimeric antigen redirected (CAR) T cells have been developed, so that they can bind to the IL13R*α*2 [100], her2 receptor [101], EGFRvIII [102] or to the ganglioside GD3 [103].  Figure 1 shows the current forms of CAR currently used for possible therapy of human gliomas. After the CAR construct is engineered, then the gene is packaged within either adenoviral or retroviral vectors. This allows one to quickly transfect as many patient's T lymphocytes as possible. When these transduced peripheral blood T cells are reintroduced back into the patient, preferably near or in the cancer inside, the CAR-redirected T cells can attack the cancer. IL-13R*α*2 based CAR/zetakine transduced T cells kill several human glioma cells *in vitro* and appear effective in intracranial gliomas in immunodeficient mice. With her2 specific CAR lymphocytes, therapeutic efficacy was seen in a xenogeneic model with the Daoy (her2+) medulloblastoma, when these human CAR T cells were adoptively transferred into these mice [104]. Her2 CAR constructed human T cells killed both CD133+ and CD133- GBM cells. Her2-redirected CAR T cells showed some efficacy against the human her2+ gliomas growing in SCID mice [101]. The advantage of using CAR-T cells is that they are also applicable towards other cancers like her2+ breast and ovarian cancers; EGFRvIII+ engineered CAR T cells can also target lung cancers; while ganglioside GD3 CAR-T cells can also interact with melanoma cells. Currently CAR T cells are being used clinically at the City of Hope (Duarte, Ca) and the clinical trial using the CAR-her2 cells is expected to start recruiting patients in the mid-late Fall of 2010 at the Center for Cell and Gene Therapy of Baylor College of Medicine (Houston, TX).

**Dendritic Cell-Based Vaccines**

Dendritic cells (DC) are currently the favorite therapeutic modality now used in cancer immunotherapy. Monocytoid dendritic cells are readily available from the peripheral blood monocytes and can be quickly activated *ex vivo* using cytokines as granulocyte-macrophage colony stimulating factor (GM-CSF) and interleukin 4 (IL-4). This technique is quite versatile in that different sources of antigens can be added to the DC. Killed tumor cells, tumor cell extracts, purified tumor mRNA, or purified tumor antigens can be given to the DC and these cells can then properly process the tumor antigens, so that the peptides are presented in the MHC. These DC are capable of immunizing naïve animals [105–107]. Plasmacytoid DC cells are beginning to be used for cancer immunotherapy [108], but so far, they have not been developed for brain cancer therapy. Once *ex vivo* activated and antigen-pulsed DC are generated, some protocols allow the non-matured DC or matured DC to be injected back into their patients. The non-matured DC are thought to become mature after reintroduction back into the patient, especially after the injection of the TLR antagonists, which act as an adjuvant to cause DC maturation.

Worldwide there are multiple centers [109–114] generating DC used for brain cancers. Clinically positive responses for usually reported for a subset of glioma patients. Usually this means that the mean time to progression for these treated patients with high grade gliomas increased in these responder populations. Kim and Liau [115] reported that their vaccine responders survived 642 ± 61 days when compared to the non-responders (430 ± 50 days). Disease free progression was also improved by 4.5 months. Some patients are reported to survive more than five years. One key finding that has been repeatedly reported is that this DC-based immunotherapy is safe with few serious side effects.

A variant of the dendritic cell-based vaccine occurs by fusing the DC with the glioma cells to form an immunostimulatory cellular hybridoma. Here the DCs are fused with the autologous glioma cell line. This strategy is analogous to the classical hybridoma used for antibody production, except the end function of this hybrid is to stimulate an immune response. The glioma parent cell supplies the correct tumor antigens, which should provoke the proper host specific immune response. The DC parental cell provides the machinery to take the glioma antigens, and process the peptides onto the patient's MHC (HLA-A, B, C, and D loci). The DC parent also supplies the costimulatory molecules and cytokines to make this hybrid an immunostimulatory cell. When these x-irradiated hybrid cells are injected back into the patient, they now provoke an immune response, so that multiple T clones responding to multiple glioma antigens are elicited. In clinical studies, Kikuchi and colleagues used this approach to treat 6 patients [116]. This first study showed this hybridoma vaccine was safe, but it failed to achieve any clinical effect. In the follow-up study, the DC hybridoma was combined with an injection with recombinant IL-12, 3, and 7 days after the hybrids were injected. This combination achieved some disease stabilization and tumor shrinkage in four out of 15 patients that were treated [117]. After one year, two of the patients still survived.

Finally, one aspect of DC that is not now fully appreciated is that IL-4/GM-CSF activated human DC can kill glioma cells [118]. Both human and rodent DC can directly kill human gliomas either by direct contact or by the release of nitric oxide. Others have previously reported this cytolytic phenomenon towards other human cancers [119, 120] by the release of type I or type II interferon and membrane cytolytic-inducing molecules (i.e., TRAIL, NKG2D, Fas Ligand). No one is currently using activated DC as the actual cytolytic effector cells against glioma. Most researchers consider the best use of DC is to stimulate T-effector cells by a systemic vaccination route.

## 3. Tumor Antigens

Identification of clinically relevant tumor antigens is actively researched. New tumor antigens seem to be reported monthly. Tumor antigens are identified by either antibodies or by T cells. The latter are recognized by T cells in the context of the TCR with either MHC class I which are recognized with the help of CD8+ molecules or MHC class II that are recognized by CD4+ molecules. Cheever and colleagues [121] have recently prioritized a set of 75 human tumor antigens, which they determined should be further developed for cancer immunotherapy. This prioritization was based on a number of key factors, such as possible therapeutic function, the immunogenicity of these molecules, their roles in oncogenesis, its specificity, and its frequency in a number of cancers. At least 18 of these listed tumor antigens are pertinent to human brain cancers.

Tumor antigens can be defined as either being tumor-specific or tumor associated. Tumor-specific antigens are actually rare, while tumor-associated antigens are expressed on normal tissue and are simply overexpressed by the tumor.  Table 1 lists the antigens that can be considered glioma-associated antigens. Surprisingly, there are really no truly glioma-specific antigens currently known. All these antigens can be found within a variety of other tumor types. But from experimental evidence, we know there is tumor specific immunity. So there are undoubtedly many glioma-specific and glioma-associated antigens still to be found.

### 3.1. Tumor-Specific Antigens

Tumor-specific antigens include p53, EGFRvIII and ras mutations. These antigens are quite common in many types of cancers. Gliomas rarely have ras mutations, but frequently possess p53 point mutations, which inactivate its normal function. Mutated p53 can be recognized by murine CTLs by wild type-p53 peptides that bind to MHC class I alleles [123]. Human CTLs responses towards p53 can also be developed in an identical fashion [124, 125]. Many cancer patients possess discernable antibody responses to p53 [126], so some Th2-mediated responses generated towards MHC class II-restricted antigens are needed to help produce these higher affinity IgG antibodies. Many glioma antigens are called “antigen recognized by T cells” (ART), for example, ART-1, ART-4, or “squamous antigen recognized by T cells” (SART); that is, SART-1, -2, and -3. These last 5 antigens were identified within either glioma cell lines or within adult or pediatric brain tumors [127].

### 3.2. Overexpressed Antigens

These antigens are found in normal cells and tissue, such as B-cyclin and CD133. These antigens appear to be overexpressed on their cancerous counterparts. Some antigens are found only within the testes and cancers. Hence the term “cancer-testes antigen” is frequently given, to describe them. Some of these antigens include Mage-1, Gage-1, SSX2, and NY-Eso-I. These antigens are found in terminally differentiated melanocytes and in their transformed progeny (melanomas) and in gliomas. Some differentiation antigens are not found in the testes, but are found on normal cells like melanocytes and in melanomas and in gliomas, with Trp-1, and Trp-2 being representative of this group. Since melanomas and glioma cells share a common embryonic neuroectoderm precursor, it is not that surprising that these two cancers share many common antigens.

### 3.3. Viral Antigens

Many viruses are thought to play a causative role in some human cancers: HTLVI, hepatitis B and C virus, EBV, and papilloma virus. Recently, Cobbs and his colleagues [62] linked cytomegalovirus (CMV) with human gliomas. CMV is frequently detected within chronically immunosuppressed patients with either transplant patients or in late stage HIV infections suffering from Acquired Immune Deficiency Syndrome (AIDS). It is thought that 70–90% of the population are previously exposed to CMV and might be chronically infected with this virus. Our immune systems keep this virus under tight control. Glioma patients are frequently considered immunosuppressed by a number of mechanisms [128]. So when the immune system is impaired as in GBM, the CMV can now reappear. Whether CMV directly causes glioma is a controversial topic. The possibility that CMV attaches itself to glioma via the PDGFR*α* allows some interesting therapies to be explored. Viruses are usually good targets for the immune system. One CMV antigen, pp65, induced human HLA-A2 immune responses in a GBM patient [129]. Freshly isolated glioma samples seem to express this antigen to a high degree [130], but cell lines lose expression of CMV. If a high number of GBM cells do attract and harbor CMV *in vivo*, then this opens up the possibility of developing CMV peptides to vaccinate against the virus and therefore the glioma is targeted indirectly. Currently, Duke University is actively using DC-based vaccines targeting CMV antigens to treat their patients who are CMV positive. Clinicians at Penn State University are using allogeneic CMV-specific CTLs to treat their glioma patients [131, 132]. The Center for Cell and Gene Therapy of Baylor College of Medicine is also developing a CTL approach against CMV as the way to treat GBM, these clinical trials will shortly begin in the next few months. Finally, another possibility is to use the CMV virus as a vector to deliver therapeutic agents or genes specifically into these gliomas to make them more vulnerable to assorted therapies. Thus, CMV may be a very useful immunological tool to attack GBM.

## 4. Source of Antigenic Materials

The choice of a source of the tumor antigen is probably the most important decision to be made when it comes to vaccinating cancer patients. There are multiple sources of antigenic material: cell lines (whole cells or lysates), fresh surgical tissue (cell lysate, mRNA, or primary cultures of tumor “stem cells” grown as neurospheres), and peptides (acid eluted or synthetic). Each choice has its own pros and cons for their clinical usefulness. The knowledge gained from these clinical studies using cell lines, neurospheres, surgical specimens, and peptides will undoubtedly advance the field, once we determine what the best source of tumor antigens is. Unfortunately, only trial and error will tell us the best source of tumor antigens for clinical responses.

### 4.1. Cell Lines

Traditionally, cell lines were used for examining immunization properties either in animals or in early clinical trials. These cells generally represent a stable and continuous source of tumor cells and they can reproducibly form cancers in experimental animals. Additionally, these cells can be modified with cytokines or costimulatory molecules to improve their immunogenicity. Cell lines created from the 1960 to 1980's are still widely used: Uppsala Sweden-derived cells (U87, U251, U373, etc.), Duke University-derived (D54, D68, etc.), Lucerne Switzerland-derived (LN18, LN 229, etc.), Surgical Neurology Branch (Bethesda, MD)-derived cells (SNB19) are still quite useful for studying various aspects of glioma biology both *in vitro* and *in vivo* within immunodeficient mice. These glioma cells readily form either intracranial or subcutaneous tumors in these immunocompromised mice. SCID/NOD mice can be “humanized” by prior transplantation of human thymus and bone marrow. One can examine human immune responses *in vivo* without doing expensive clinical trials, while quickly exploring the feasibility of generating these human-specific responses. 

Established cell lines can either be used as whole-cells or as a lysate to be the immunogen. Cell lysates can be combined with adjuvants or it could be used as an irradiated whole cell. Cell lines can be genetically modified with cytokines or costimulatory molecules to improve the immunogenicity of the cells. Parney and colleagues [133, 134] have shown this possibility of genetically engineered glioma vaccines within immunodeficient mice. 

Zhang and colleagues [135], examined 20 human GBM cell lines, some well-established cells such as U87, U251, D54, LN18, SNB19, and so forth, along with some recently derived cells (NovaRx: NR203, 206, etc.). These glioma cell lines were characterized for their tumor antigen expression of 20 tumor-associated antigens by quantitative reverse-transcriptase real-time polymerase chain reactions (qRT-PCR) techniques. The translated proteins were confirmed by immunoflourescent antibody staining and intracellular flow cytometry for 16 tumor antigens, since those antibodies were available. With the exception of 3-4 antigens, the cell lines were all quite homogenous and had high tumor-associated antigen mRNA expression. Hence a cell line or combination of cell lines can easily be used as a universal source of antigenic material for any potential vaccine. Besides the currently known tumor-associated antigens (Table 1), the other advantage is that cell lines can also be a source of currently undiscovered antigens. Zhang et al. [127] showed that surgical specimens derived from adults with GBM were highly antigenic (29 antigens were routinely expressed), while the surgical specimens derived from pediatric GBM, ependymoma, pilocytic astrocytomas were more restricted in their tumor antigen profile (9–16 antigens).

Cell lines grow quite easy and can be produced in bulk in an economic manner. To use them as a clinical product, one needs to do extensive clinical testing for all types of pathogens (mycoplasma, bacteria, fungi and viruses). Some safety testing takes the cells/cell lysates and exposes them to other human/animal cells, injected into suckling mice, guinea pigs, and chicken eggs to assure no cryptic pathogens are present when grown in a more permissive environment. As expected this procedure is quite expensive ranging from US $50–80 K/cell line. This procedure is required, if the vaccine material from one person is being injected into a different patient. But if the autologous cells are custom-made for that given patient, then this added safety test usually is not required for the exotic viral pathogens. Only the common microbial contaminants need to be tested. This financial restraint helps explain why most current protocols are using autologous gliomas. A cell lysate is made directly from the resected surgical specimen, so the risk is minimized for possible microbial contamination. Currently two companies, NovaRx (San Diego, CA, USA) and Epitopoietic Research Corporation (ERC, Gembloux Isnes, Belgium) are developing allogeneic-based vaccines. NovaRx is developing whole cell line vaccines by knocking down the glioma's ability to make TGF-*β*. Thus, shutting down the ability of these cells that lead to immunosuppression, and this leads to improved in vivo responses [136]. This irradiated whole-cell-based vaccine is known as Glionix. The Glionix has been safely used to vaccinate six patients and the results were encouraging, so that further Phase II/III studies are currently being initiated. ERC is developing a cell lysate approach by combining tumor lysates from a number of GBM specimens. This company is currently accumulating glioma tissue to be used for their cell lysate-based vaccine.

An early success using cell lines as a source of tumor vaccines was reported in the early 1980's when the Duke University group used D54 and U251 cell lines to vaccinate human glioma patients [137]. Some vaccinated patients became long-term survivors after being vaccinated with these irradiated whole cells. The use of the killed U251 (HLA-A2+) cells was reported to be the most effective, when compared to the D54 (HLA-A3+) vaccine. Unfortunately, this study proved to be premature and illustrated the “growing pains” of the field. It is now felt that patient selection, such as using patients with lower grade gliomas, better prognosis status, or younger patients with a better survival prognosis (proneural GBM subtypes) were used with the U251-based vaccine. 

The human glioma cells used for that clinical vaccination were cultured in fetal calf serum (FCS), which is routinely used for tissue culture. When the vaccinated patient's sera were tested, most patients had antibodies against the bovine proteins found in FCS [138]. Antibodies against the immunizing glioma's HLA antigens were additionally found. One patient had an undefined antibody specific for the U251 cells. This work clearly showed that humoral immunity was induced within these patients by using an irradiated whole cell vaccine. Whether the FCS acted as a xenogeneic adjuvant or as a mechanism of “epitope spreading” [139] is an intriguing possibility.

### 4.2. Stem Cell Lines

The discovery of proper cell culture techniques for growing “stem cells” or “cancer initiating cells” as neurospheres has helped advance the field of glioma biology. Here surgically removed tumor cells are dissociated and grown in serum-free media containing epidermal growth factor and basic fibroblast growth factor [140]. The concept of “glioma stem cell” has emerged as a hot topic. Many of the features seen in clinical glioma are better reproduced when the CD133+ stem cells, rather than those coming from established cell lines are injected into immunodeficient mice. Only 10–100 of these cells are needed to form tumors in these mice within 30–60 days. The resulting gliomas derived from CD133+ cells display an invasive phenotype, as opposed to well-circumscribed borders that form when established (differentiated) cell lines are used. GBM stem cells can also explain why gliomas resist certain drugs and radiation [141, 142]. CD133 was initially described as a marker which resisted the uptake of fluorescent markers, which coincidentally resemble chemotherapeutic drugs [143]. However some GBM stem cells are also reported as being CD133-negative [144]. So the whole concept of GBM stem cells is still being refined.

Glioma stem cell lines have been used as a vaccine in rodent models and appear safe and effective to vaccinate against some rodent gliomas [145]. Some human glioma stem cell lines have been characterized for their tumor antigen profile [146]. With the exception of CD133 for some stem cells, GBM stem cells possess few truly tumor-specific antigens. So it is unlikely we will be able to specifically only target stem cells by immunotherapy. Tumor associated antigens such as TRP2, GP100, EGFR, AIM2, and Sox2 are present on these human glioma “stem” cells. In contrast, IL-13R*α*2 and her2 seems to be diminished in these human stem cells.

### 4.3. Surgical Resected Lysates

Tumor lysates are most frequently used, clinically. After the patient recovers 6–8 weeks from debulking surgery, the patient is leukopheresed to acquire sufficient DC precursors. The debulked tumor was collected, analyzed and aliquots are saved for vaccinating protocols with the DCs. This process is relatively simple and straightforward with minimal risk of contamination, as opposed to long-term cell culturing. 

When glioma surgical samples (WR-GBM) were injected with Freund's adjuvant (complete or incomplete) into monkeys and guinea pigs, an autoimmunity resembling experimental allergic encephalitis (EAE) was produced [147]. However, when the glioma cell line that was derived from WR-GBM (D-68) was used as the vaccine without Freund's adjuvant, no autoimmunity was seen. As a result of these potential autoimmune complications, vaccination using glioma surgical samples was largely abandoned and future attempts were usually discouraged by citing this case. The results using DC pulsed with surgically resected tissue have been shown to induce tumor immunity without any signs of EAE [109–115]. This empirical evidence also overcame the prejudice that was initially elicited from that prior EAE induction paper [147].

So what are the best sources of antigenic material coming from cellular sources? Since cell lines and stem cell neurosphere cultures are pure tumor cells, it would be expected that these sources would contain the most tumor-associated antigens, while the surgical specimens are a mix of tumor cells and normal hosts cells like: endothelial cells, neurons, microglial, and other hematopoietic cells that were present within the tumor. These normal cells will therefore dilute out the tumor antigens coming from the tumor cells. This is potentially significant, because when one uses this material to pulse dendritic cells, irrelevant normal host antigens may be loaded onto the binding grooves of the MHC molecules, hence stimulating the immune system to a lower extent. Since there is a finite number of MHC molecules per dendritic cell, loading irrelevant peptides may make the immunostimulatory process to T cells less efficient. 

The concept of vaccinating against the tumor's vasculature was reported [148]. The late Judah Folkman [149] used to argue that the endothelial cells of tumors are normal cells. These normal endothelial cells would therefore not be subject to the same mutational rates as cancer cells. So these cells would make the best target for cancer therapy, rather than directly targeting the cancer cells. Virrey et al. [150] have found that endothelial cells derived from gliomas are different than those endothelial cells derived from the normal noncancerous brain, these cells have morphological changes and grow slower than expected. Two recent studies suggest that GBM “stem” cells can differentiate into glioma endothelial cells [151, 152]. So there could be legitimate reasons for using the whole tumor lysate as a vaccine in order to target these abnormal endothelial cells. May be some of the success seen, when the tumor cell lysate was fed to the DC was due to immune responses directed towards the glioma's vasculature. This open question is an important issue that will be needed to be answered before the next major advance towards cancer immunotherapy is made by using cell-based materials.

### 4.4. Peptides

#### 4.4.1. Peptides Vaccines

A more refined approach is to use possible antigenic peptides as the starting vaccine. One knows exactly how much antigen is given to the DC, as opposed to cells or tumor lysates. Liau and her coworkers [153, 154] and Yu and his colleagues [155] used this strategy to pulse their dendritic cells. Here tumors or tumor cell lines were acid eluted and the extracted peptides were loaded onto the patient's DC. Some T cells stimulated with this method did generate T cells that infiltrated the recurrent glioma. Liau et al. [153] did use peptides derived from allogeneic glioma cell lines and then pulsed the patient's DC with these peptides. Little evidence of beneficial antitumor immunity was seen. This might explain why these DC researchers quickly switched to using the tumor cell lysates obtained from surgical specimens as the starting vaccine material.

The Duke group is using synthetic peptides-based strategies to vaccinate EGFRvIII mutated GBM [156, 157]. The University of Pittsburgh (Pittsburgh, PA) is using glioma associated peptides (Survivin 96–104(2M), WT1 126–134(1Y), EphA2 883–891, and IL-13R*α*2 345–353(1A9V)) along with poly ICLC to vaccinate their patients [158]. One of the advantages of using synthetic peptides, is that one can design peptides that have a higher binding affinities for the MHC molecules than the actual tumor-derived peptides. This is evidenced by the use of redesigned survivin, WT-1 and IL-13R*α*2 peptides that the Pittsburgh group is using. Currently, the NYU Pediatric Neuro-Oncology Group in collaboration with their Melanoma-DC program is treating patients with the autologous pediatric DC with several peptides (gp100, TRP2, EphA2 and her-2) for various pediatric brain cancers (low grade gliomas, GBM, ependymoma, and medulloblastoma). To date, they have vaccinated four children without any signs of toxicity (Dr. Sharon Gardner, personal communication). Since children survive longer with brain cancers that their adult counterparts (CBTRUS, 2010), it will take some more time before the actuarial data is collected before we know whether this therapy worked.

Of course, one restriction using specific peptides is that these patients have to be HLA-matched to assure that the peptides will properly bind to the patient's own MHC. Roughly, half of population in the USA have the HLA-A2 allele, while Japan and China have higher proportions of HLA-A24 allele, so most studies will need to be based on their correct HLA-alleles. It remains to be seen whether better clinical responses will be generated with these peptide-based vaccines when compared to the cell lysate pulsed DC. One possibility is to use the entire tumor-associated antigen precursor protein as opposed to small peptides to pulse the DC. Here the DC will process the proteins so that the correct peptides will bind to its own unique MHC molecules. So the patient's own DC will customize their proper antigenic peptides to fit with their own immunogenetics.

### 4.5. Nongenetic Manipulated Vaccines

Cells can die by at least three distinct pathways: apoptosis, autophagy, necrosis/paraptosis. Bredesen et al. [159] and Hotchkiss et al. [160] have reviewed the biological processes involved in these different pathways possess. Each pathway has its own unique ability to interact with the immune system. These types of vaccines could be used either with cell lines or the stem cells.

#### 4.5.1. Apoptosis

Radiation and most chemotherapeutic agents kill tumor cells by initiation of apoptosis [161, 162]. In response to these treatments, many *in situ* tumors initially shrink and regress, only to reoccur with a more malignant phenotype sometime later. Despite a massive release of tumor-specific material, including tumor antigens, no lasting immunity or tolerance occurs [163, 164]. Apoptosis has been called the “silent death” and does not usually provoke immunological responses. Most apoptotic cell remnants are taken up by adjacent cells. Apoptosis is the driving pathway that induces immune tolerance towards many self-antigens either in the thymus for T cells or in the bone marrow for B cells. The only way to override this immunotolerizing property of apoptotic cells is use some immunostimulatory cytokines like either IL-4 or GM-CSF [165, 166] or by costimulatory molecules which help stimulate the APC function [134].

#### 4.5.2. Autophagy

This process occurs when cells are stressed or deprived of key nutrients. These affected cells undergo a process whereby they begin to self-digest themselves. The key morphological change in autophagic cells is having double membraned vesicles within the cell. Like apoptotic cells, autophagic cells do not induce *in vivo* inflammatory responses. Autophagy may be a way that cells infected with intracellular pathogens commit suicide, thereby limiting the intracellular infection. Breast cancer cells treated with 4-hydroxytamoxifen can be stimulated to undergo autophagy. So autophagy may be useful for certain clinical therapies. However some breast cells survive this therapy and then show drug resistance [167]. Under *in vitro* conditions, autophagic tumor cells can be fed to dendritic cells and produce T-effector cells [168, 169].

#### 4.5.3. Necrosis/Paraptosis

The mechanism of necrosis-induction is the least well-defined pathways of the three forms of programmed cell death. It is thought that paraptosis is the programmed pathway that leads to necrosis. Paraptotic cells are characterized by a swelling and vacuolization process that starts with the physical enlargement of the endoplasmic reticulum (ER) and mitochondria [170]. Swollen cells suggested that ionic disregulation is accompanied by water influx and retention. The disruption of intracellular ion homeostasis ultimately causes these cells to osmotically lyse releasing intracellular contents, such as high gel mobility binding protein-1 (HMGB1) [171], heat shock proteins [172], and various proteases. These proteins act as “Danger Signals” promoting massive inflammation and cellular immunity [173]. Hence, the best way to improve the natural immunogenicity of the tumor cell is by using necrotic/paraptotic cells. In contrast, vaccination with necrotic tumor cells produces superior T-cell immune responses in comparison to those responses elicited by immunization with apoptotic cells [163, 164, 174]. The advantages of using the tumor's own natural death pathway allows for the autologous glioma cells to be used as a vaccine without having to do any genetic manipulations, which could delay the time that a vaccine could be given to the patient with a relapsing glioma.

Over the last decade our lab pioneered the use of genetically engineered tumor cells with membrane M-CSF as a tumor vaccine (mM-CSF, 38–41). Activated macrophages killed these mM-CSF transduced tumor cells quite easily in at least four different tumor models (rat glioma, human glioma, mouse hepatoma [175], and rat breast cancer [176]). In our glioma models (rat T9 and human U251), the mM-CSF+ tumor cells are killed upon binding by the responding monocytes followed by the release of reactive oxygen species (ROS). The interaction with ROS resulted in paraptosis [49, 177–179]. 

The limitation of using mM-CSF-based glioma vaccines was that this tumor vaccine required living cells in order to produce the immunogenic stimuli needed for the vaccine. If the mM-CSF transduced T9 cells were either x-irradiated, mitomycin-C or freeze-thawed prior to subcutaneous injection, the vaccinating effects of mM-CSF+ glioma cells would be lost [40]. Hence no IRB or study section would permit a living tumor cell vaccine to be used in human patients. So the mechanism by which these paraptotic cells were induced, was investigated. This would then reproduce the same immunogenicity of mM-CSF+ cells but now with killed glioma cells. By forcing open BK (big potassium ion channels) with phloretin or pimaric acid, paraptosis was induced within the glioma cells along with the increased production of heat shock proteins (HSP60, HSP70, HSP90, and Grp94) and the peripheral migration of HMGB1 to the cell surface. When these cells now osmotically lyse, all these “danger signals” are now released and available to stimulate the local APCs. All vaccinating effects of living mM-CSF+ tumor cells can now be reproduced by using our killed BK channel activated/killed glioma cells. All gliomas (rat or human) that we tested have been successfully killed by prolonged exposure to BK channel activators [175, 176]. So this method can be developed for possible clinical trials.

## 5. Summary

Progress towards brain cancers by using immunotherapy is slowly moving forward. Initial attempts used nonspecific approaches like adjuvants and LAK cells. Nonspecific cellular approaches were only effective for a small minority of fortunate people. The general focus now is directed towards specific methods. These specific humoral methods include using monoclonal antibodies and scFV fragmented antibodies. Specific cellular approaches include using TILs/CTLs, alloreactive CTL stimulated by MLRs, all appear to have generated some clinical success. Active immunization with autologous DCs that have been loaded with tumor-antigens also appear to generate long-term survivors. Glioma cells seem to possess numerous tumor-associated antigens. Identification of other strategies that can be combined with immunotherapy approaches will certainly improve our success against these lethal brain cancers. We look forward towards the next chapter of this story as the field continues to mature.

## Figures and Tables

**Table 1:** List of tumor-associated antigens known within human brain cancers.

Aim-2	Art-1	Art-4	B-cyclin	CD133	EGFRvIII
Epha2	Ezh2	Fosl1 (fra-1)	Gage-1	Galt-3	Ganglioside GD3
Gp100	GnT-V	Her2	HNRPL	IL-13R*α*2	Livin
Mage-A1	Mart-1	MELK	MRP-3	NY-Eso-1	Prame
PTH-rP	Sart-1	Sart-2	Sart-3	Sox 2	Sox10
Sox 11	SSX-2	Survivin	Tert	TRP-1	TRP-2
Tyrosinase	Ube2V	Whsc2	WT-1	YKL-40	
SLC01C1*	BCAN*	CHI3LI*	CLIP2*	FABP7*	NR2E1*
NLGN4X*	NES*	NRCAM*	PDPN*		

Asterisk denotes potential tumor antigens described in [122].
